# Remote Gaze Tracking System on a Large Display

**DOI:** 10.3390/s131013439

**Published:** 2013-10-07

**Authors:** Hyeon Chang Lee, Won Oh Lee, Chul Woo Cho, Su Yeong Gwon, Kang Ryoung Park, Heekyung Lee, Jihun Cha

**Affiliations:** 1 Division of Electronics and Electrical Engineering, Dongguk University, 26 Pil-dong 3-ga, Jung-gu, Seoul 100-715, Korea; E-Mails: leehc@dgu.edu (H.C.L.); 215p8@hanmail.net (W.O.L.); cho4400@dgu.edu (C.W.C.); gwonsuyeong@dgu.edu (S.Y.G.); 2 Electronics and Telecommunications Research Institute, 218 Gajeong-ro, Yuseong-gu, Daejeon 305-700, Korea; E-Mails: lhk95@etri.re.kr (H.L.); jihun@etri.re.kr (J.C.)

**Keywords:** remote gaze tracking, large display, WVC and NVC, panning and tilting

## Abstract

We propose a new remote gaze tracking system as an intelligent TV interface. Our research is novel in the following three ways: first, because a user can sit at various positions in front of a large display, the capture volume of the gaze tracking system should be greater, so the proposed system includes two cameras which can be moved simultaneously by panning and tilting mechanisms, a wide view camera (WVC) for detecting eye position and an auto-focusing narrow view camera (NVC) for capturing enlarged eye images. Second, in order to remove the complicated calibration between the WVC and NVC and to enhance the capture speed of the NVC, these two cameras are combined in a parallel structure. Third, the auto-focusing of the NVC is achieved on the basis of both the user's facial width in the WVC image and a focus score calculated on the eye image of the NVC. Experimental results showed that the proposed system can be operated with a gaze tracking accuracy of ±0.737°∼±0.775° and a speed of 5∼10 frames/s.

## 1. Introduction

Recently, intelligent televisions such as the Internet protocol television (IPTV) and the smart TV have been widely popularized. The intelligent TV provides not only conventional broadcasting but also many services such as video on demand (VOD), web surfing, shopping, teleconferencing, and social network services (SNS) [1]. This development has caused the conventional remote control to become exceedingly complex because many additional buttons have been included to perform various functions on the remote control [2]. In addition, people must learn what functions are performed according to the type of buttons on the remote control. This issue adversely affects user convenience. In order to solve these problems, we propose a novel gaze tracking system as an intelligent TV interface for a large display.

Gaze tracking is a technology used to detect where a user is looking, and gaze tracking systems have generally been divided into two categories in previous studies. The first category is a wearable gaze tracking system, and the second is the remote gaze tracking system [3]. In the case of a wearable gaze tracking system, a user must wear a device that includes an eye-tracking camera [3–9]. In case of a remote gaze tracking system, a user does not need to wear any device, and the user's eye image can be acquired by a remote camera system [1,10–19]. Although the former system has advantages in terms of accuracy and system complexity, the user inconvenience of the former system is higher than that of the latter one because of the need to wear the device.

In previous studies, gaze tracking was performed at a close distance (less than or equal to 1 m between the camera and the user's eye) [3–9,13–15,19]. Desktop monitors are commonly used for testing gaze tracking methods, with a small screen. However, some researchers have studied gaze tracking systems that are employed at distances greater than 1 m, with limitations [1,16]. Their systems could not cover a sufficiently wide range to allow for free head movement of the user. In the previous research [1], although they made remote gaze tracking system for IPTV at a distance, their camera system does not include the functionality of panning, tilting and focusing, so a user's movement is allowed within a limited range of X, Y and Z.

There have been other previous studies of long distance gaze tracking (*i.e.*, greater than 1 m between the camera and the user's eye). Yamazoe *et al.* proposed a remote gaze tracking method that uses a single camera at a distance of 220 cm, but the gaze detection error was significantly high (on an average, the error was 5.3° horizontally and 7.7° vertically) [11]. Zhu *et al.* proposed a remote gaze tracking system by using active infrared (IR) lights at a distance of 1–1.5 m. However, the errors of their system were also considerable (almost 5° horizontally and 8° vertically) [12]. Several companies such as Tobii [17] and Eyetech [18] have manufactured commercial gaze tracking systems that can be used at a long Z distance. However, the freedom of head movement of the user in front of the eye tracking system is limited, and these systems have the disadvantage of being large.

In previous research [20], an interaction method between large screens and personal devices with gaze and touch was proposed. However, the freedom of the user's head movement is limited in front of the gaze tracking system. In other previous research [21,22], the authors proposed a gaze tracking system at a distance, which includes two narrow-angle cameras having the functionalities of panning and tilting, and two wide-angle cameras by which the Z distance between the system and user's eye is measured. However, by requiring a total of four cameras, their system has the disadvantage of high cost and large size. In addition, their system requires the additional procedure of camera calibration. Corcoran *et al.* proposed a gaze tracking method using a visible light camera [23], but the freedom of user's head movement is limited in front of the gaze tracking system. In [24] a long range eye tracking system based on the narrow-angle camera having the functionalities of panning and tilting was proposed, but the system requires an additional device (a Kinect device from Microsoft Corporation) which provides the wide-angle camera image and depth information by which the panning and tilting of the narrow-angle camera are performed. Zhang *et al.* proposed a gaze interface system using a visible light camera on a 55 inch LCD display [25]. However, they positioned the gaze tracking camera close to user's face (60 cm in front of the display), and it can be inconvenient to the user by hindering the user's natural line of sight. In [26], the authors proposed a mobile gaze-based screen interaction system on a 55 inch TV, however, they use a wearable gaze tracking device which a user must put on, which is inconvenient for the user. In general, gaze tracking for an intelligent TV with a large screen is different from that for a desktop computer, and the following factors should be considered:

(1) Because the Z distance between a user and a TV is greater than that of a desktop monitor, considering the conditions of TV viewing, even a small error in gaze estimation can produce large pointing errors on a TV screen.
(2) Because the conventional duration of a TV viewing session is typically longer than that of a session using a desktop computer, the inconvenience of any gaze tracking device for a TV should be minimized.
(3) Complicated calibration procedures among cameras, a TV screen, and a user are not acceptable for a conventional TV viewer.

Considering these factors and to overcome the problems of previous studies, we propose a novel gaze tracking system using panning, tilting, and focusing mechanisms. Our proposed gaze tracking system was designed to be available in the range from 1.4 m to 2.7 m. Additionally, our system has a significantly wider range of freedom for the head movement of the user than a conventional remote gaze tracking system, which is suitable for a user who watches intelligent TV.

The remainder of this paper is organized as follows: an overview of the proposed gaze tracking system and its methods are explained in Sections 2 and 3, respectively. The experimental results are presented in Section 4. Finally, the conclusions of this research are discussed in Section 5.

## 2. Proposed Gaze Tracking System

### 2.1. Overview of the Proposed Gaze Tracking System

**Figure 1.**
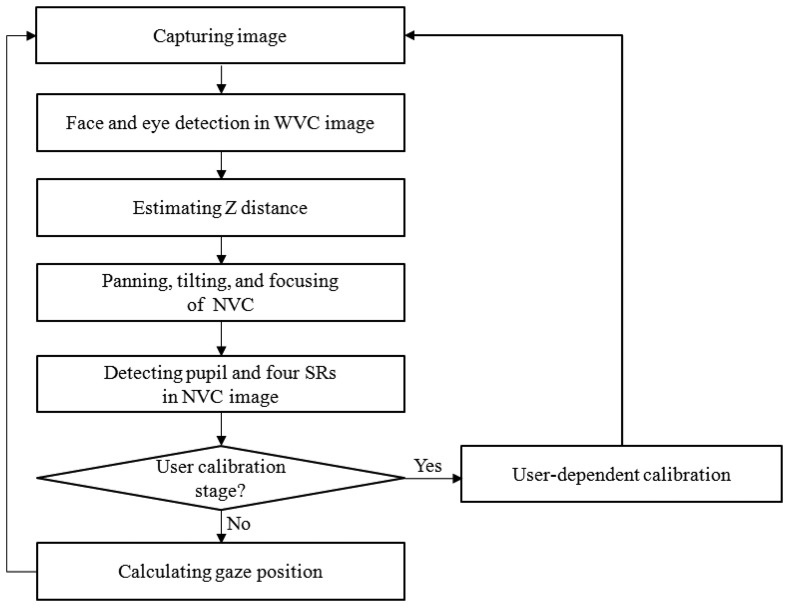
Flow chart of the proposed gaze tracking system.

The overall procedure of the proposed gaze tracking system is shown in Figure 1. When the proposed system starts, the user's image is captured by a wide view camera (WVC). Then, the face and the eye are detected in the captured image, and the X and Y positions of the user's eye can be obtained in the WVC image (see Section 3.1). In addition, on the basis of the detected face's width, the Z distance between the proposed gaze tracking device and the user is estimated in the captured image by the WVC (see Section 3.2). Then, the NVC of the proposed gaze tracking device captures the user's eye by panning, tilting, and focusing using the information of the X, Y, and Z positions of the user's eye (see Section 3.3). From this information, an enlarged and focused eye image can be obtained by the NVC, and the pupil and four specular reflections (SRs) are detected in the image captured by the NVC (see Section 3.4). For accurate gaze tracking in this study, user-dependent calibration is performed, which includes two sub-steps of focus-calibration and gaze-calibration (see Section 3.5). In the first step, user-dependent information regarding the focus is obtained [27]. In the second step, angle kappa is calculated, which is the difference between the pupillary and visual axes [1,3,5]. If the user-dependent calibration stage is executed, the gaze position of the user is calculated by using the detected pupil center and the four specular reflections on the eye (see Section 3.6). More detailed explanations are provided in Section 3.

### 2.2. Proposed Gaze Tracking Device

**Figure 2.**
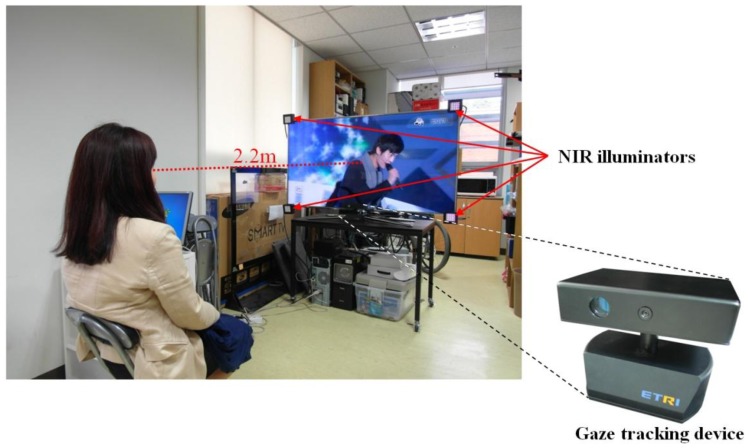
Example of using the proposed gaze tracking system with an intelligent TV.

Figure 2 shows an environment for gaze tracking with an intelligent TV with a diagonal length of 60 inches at a certain distance. The proposed gaze tracking device is located in front of and below the IPTV. In order to obtain the NVC eye image, which is illuminated only by the near infrared (NIR) illuminators, a visible light passing filter of the NVC is removed and an NIR passing filter is incorporated in the NVC [1,3–5]. This filter was incorporated because the pupil is not easily discriminated from the iris by visible light in Asian people, who have dark-colored eyes. In addition, a great deal of noise from external visible lights can be excluded in the NVC camera by the NIR passing filter and the NIR light. Four near infrared (NIR) illuminators are attached at the four corners on the TV.

**Figure 3.**
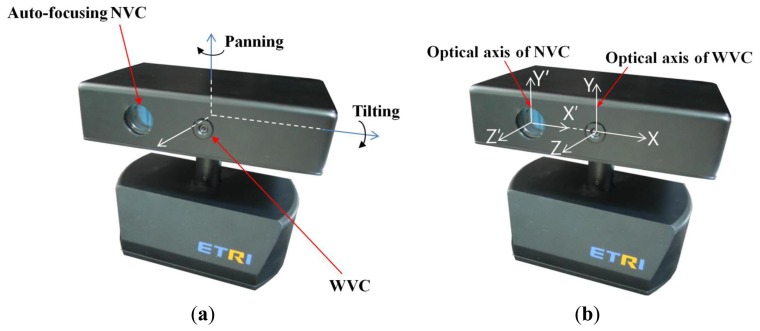
Proposed gaze tracking device. (**a**) Panning and tilting structures. (**b**) Parallel structure of the NVC and WVC.

Figure 3 shows the proposed gaze tracking device. The gaze tracking device is composed of the WVC, NVC, and three motors. The two motors are used for the panning and tilting shown in Figure 3a. The other motor is used for the auto-focusing of the NVC shown in Figure 3a.

**Figure 4.**
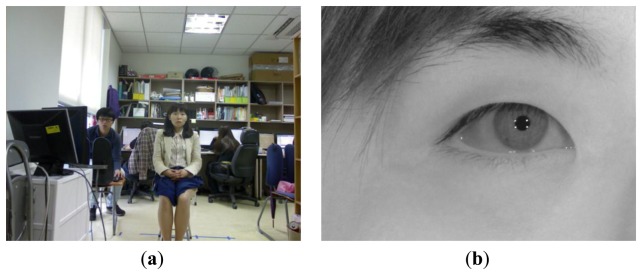
Captured images by the proposed device. (**a**) Captured image by the WVC. (**b**) Captured image by the NVC.

The WVC can capture the user in a wide field of view (FOV) and has an image resolution of 1,280 × 1,024 pixels, as shown in Figure 4a. The ranges of the viewing angles are approximately ±33° on the horizontal axis and ±25° on the vertical axis. The NVC can capture an enlarged eye image with an image resolution of 1,600 × 1,200 pixels by using a lens with high magnification, as shown in Figure 4b. In order to reduce the system size and weight of the panning and tilting motors, commercial web cameras (Logitech C600 [28]) are used for the WVC and NVC. Because the NVC will capture the magnified eye image at a distance, an additional zoom lens with a variable focal length and f-number of 10 is attached to the NVC.

Figure 4 shows the images captured by the proposed device. Figure 4a shows an image captured by the WVC at a distance of 2.2 m from the intelligent TV. As shown in Figure 4a, because the WVC has a wide viewing angle, it can capture the user's face in various positions in front of the TV. Figure 4b shows the enlarged eye image captured by the NVC on the basis of the X, Y, and Z positional information of the user's eye in Figure 4a. Thus, the four SRs in the eye image can represent the four corners of the TV screen, as shown in Figure 4b, and the user's gaze position can be calculated on the basis of the positions of four SRs and the pupil center (see Section 3.6) [1,3,5]. In the previous studies [1,3,5,6], researchers used NIR illuminators whose wavelengths were less than 900 nm, which can be seen by the user and can be inconvenient. In order to solve this problem, four NIR illuminators that have a peak wavelength of 940 nm are used in the proposed gaze tracking system.

Because the horizontal axes of the NVC and WVC are identical, as shown in Figure 3b, if we can position the detected eye in the center of the WVC in the vertical direction by tilting, we can acquire the eye image with the NVC with only additional panning. This is an advantage of the proposed parallel structure of the NVC and WVC.

The three motors used in the proposed device are controlled by its controller, which is based on an RS-232 serial interface. Because the three motors are stepping motors, the motors can be delicately rotated incrementally by a digital pulse signal. Additionally, we can adjust the steps per rotation by using motor drivers. The panning and tilting motors had a rotation rate of 1,600 steps per rotation. Furthermore, the motors have gears that are connected between its rotational axes and output axes. Thus, the panning and tilting axes can be moved by 0.025522° and 0.023864° by one pulse, respectively. This means that the panning and tilting axes have a precision of approximately 0.089 cm (tan (0.025522°) × 200 cm) and 0.083 cm (tan (0.023864°) × 200 cm) per a pulse at a distance of 2 m, respectively.

**Figure 5.**
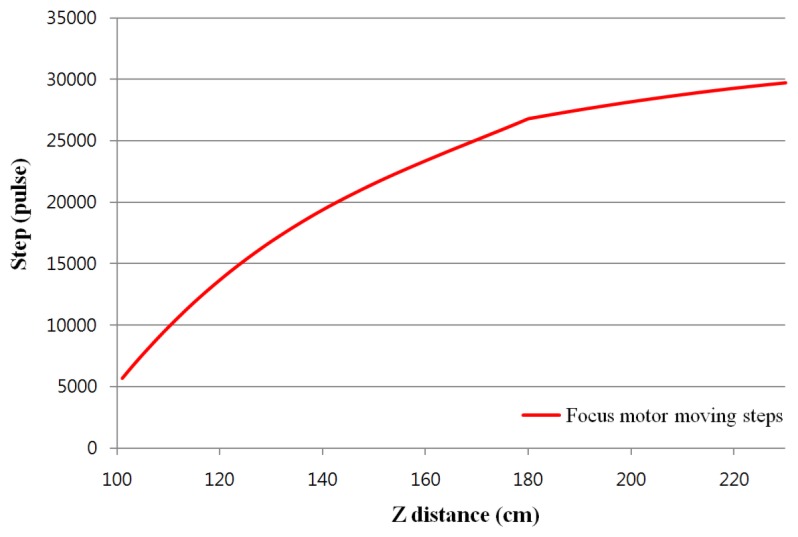
Step increments of motor for auto-focusing according to Z distance.

For focusing, we constructed a look-up table for step distance according to the Z distance, as shown in Figure 5. In Figure 5, the steps according to the Z distance were manually measured by incrementing in steps of 1 cm. The focus motor in the proposed device can be operated on the basis of this focus look-up table.

## 3. Proposed Gaze Tracking Algorithms

The proposed gaze tracking algorithms are explained in more detail in this section.

### 3.1. Face and Eye Detection in WVC

**Figure 6.**
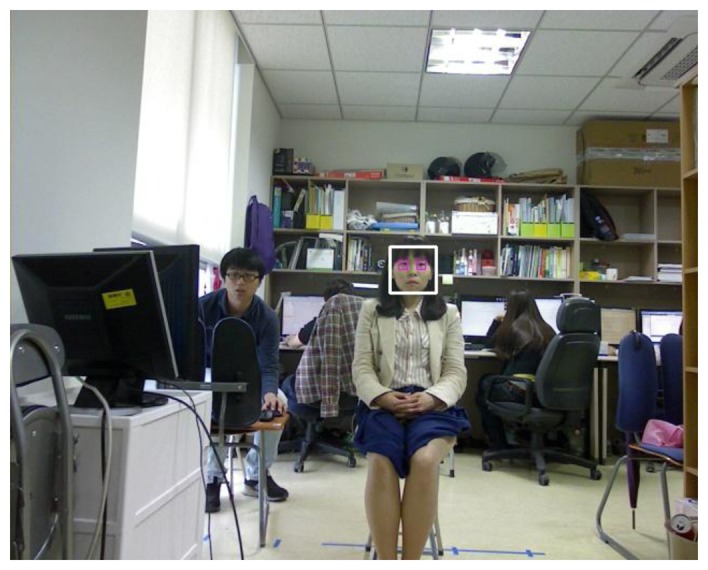
Results of face and eye detection in captured image (Figure 4a) by the WVC.

In the first step, the face and the eye are detected in the image captured by the WVC, as shown in Figure 1. To detect the face and the eye positions of the user, an adaptive boosting (AdaBoost) algorithm is used [29]. The AdaBoost face detector is well known for being able to detect the human face in a computer vision field based on Haar-like features and multiple weak classifiers [29]. Within the pre-determined area inside the detected face region, eye detection by the AdaBoost algorithm is performed. The results of face and eye detection are shown in Figure 6.

In general, the AdaBoost algorithm has limitations when detecting a rotated face. In addition, the accuracy of eye detection by the AdaBoost method can be degraded in the case of a closed eye or an eye with a glasses frame. In order to solve these problems, we combine the AdaBoost algorithm and the continuously adaptive mean shift (CAMShift) algorithm [30] to detect the face. CAMShift is an algorithm based on the histogram of an image. It can quickly track a face and is robust against variations in illumination. If the AdaBoost method fails to detect the face region, CAMShift-based face detection is performed. In order to reduce the change in illumination of the WVC image, illumination compensation using the functionality of the auto-exposure of the camera is performed.

The input image of 1,280 × 1,024 pixels is reduced in size to 640 × 480 pixels in order to enhance the face detection processing speed. With the detected face region, eye detection is performed by combining the AdaBoost algorithm and the adaptive template matching (ATM) method. Because information of precise eye position is required for accurate panning and tilting of the NVC, eye detection is performed in the original input image of 1,280 × 1,024 pixels within the eye searching area, which is defined on the basis of the detected face region. Similar to the procedure for face detection, if the AdaBoost method fails to detect the eye region, ATM-based eye detection is performed. In order to reduce the change in illumination in the NVC image, illumination compensation is performed by changing the pixel value on the basis of the average mean value of the image.

### 3.2. Estimating Z Distance

After face and eye detection, Z distance estimation is performed. For higher accuracy of gaze detection, the accurate detections of the pupil center and centers of corneal SR are required, and a focused eye image provided by the NVC is inevitably necessary. In general, when the camera captures a magnified image, the depth of field (DOF) of the camera lens becomes smaller. The DOF represents the Z distance range where a focused image can be obtained. Because the NVC of our system acquires a magnified eye image, as shown in Figure 4b, the DOF is small; consequently, the Z distance between the camera and the user is required for accurate auto-focusing of the NVC.

**Figure 7.**
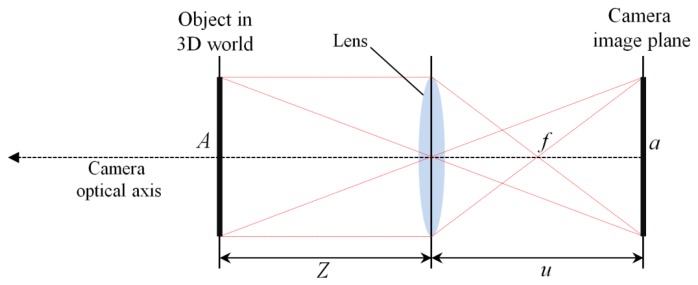
Camera optical model.

Figure 7 shows a general camera optical model that is called the thin lens model [31,32]. Variable *u* is the distance between the lens and the image plane, and *Z* is the distance between the lens and the object in the 3D world.

The object (*A*) in the 3D world is projected onto image *a* on the image plane through the camera lens. In this study, *A* is the facial width, and *f* is the focal length of the lens. On the basis of the thin lens model, the following equation is obtained [31–34]:

(1) $$\frac{1}{f}=\frac{1}{Z}+\frac{1}{u}$$

Equation (2) shows the derivation of *u* in terms of *Z* from Equation (1) [31,33]:

(2) $$u=\frac{fZ}{Z-f}$$

From Figure 7, the relationship between *A* and *a* can be represented, as shown in Equations (3) and (4) [31,33]:

(3) A:Z=a:u

(4) $$\begin{array}{ll} Za=Au, & a=\frac{Au}{Z} \end{array}$$

By inserting Equation (2) into Equation (4), we can derive Equation (5) as follows [31,33]:

(5) $$Z=\frac{Af}{a}+f$$

**Figure 8.**
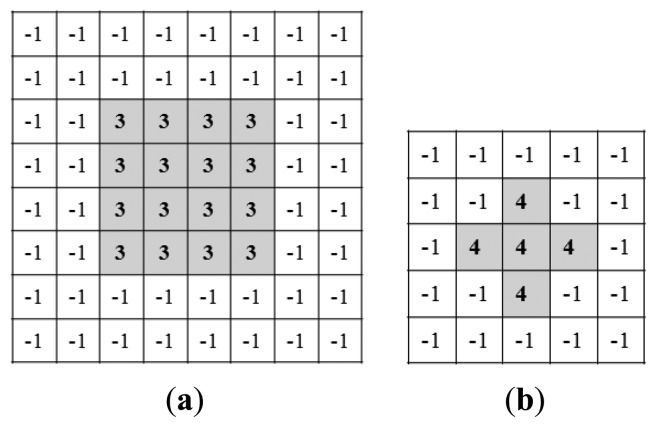
Convolution kernels for measuring focus score. (**a**) Daugman's kernel [27]. (**b**) Kang's kernel [35].

Because the WVC has a camera lens of fixed focal length, *f* is constant. Therefore, through the stage of initial camera calibration, we obtained the constant value of *f* from Equation (5). In addition, at the initial user-dependent calibration stage, the Z distance (*Z* of Equation (5)) between the user's eye and our device including the WVC can be obtained by moving the focusing lens of the NVC and by checking the focused position. At each movement step of focusing lens, an image is captured by the NVC, and its focusing condition is evaluated by using the focus mask shown in Figure 8a. When a high focusing score for a captured image is calculated by the focus mask, we obtain the position of the focus lens of the NVC, and consequently, the Z distance can be obtained on the basis of the relationship between the focus lens position and the Z distance of Figure 5 [31]. In addition, we can obtain the width of the face (*a* of Equation (5)) in the image plane at the stage of initial user-dependent calibration. Thus, in Equation (5), we know three parameters (*f*, Z, *a*) at the stage of initial user-dependent calibration, and the actual width of the user's face (*A*) can be obtained using Equation (5). During the operation of the proposed system after the stage of user-dependent calibration, because our system knows the actual width of the user's face (*A*), focal length (*f*), and facial width (*a*) in the image, the Z distance to the user can be calculated continuously by using Equation (5) [31]. However, the accuracy of the Z distance measurement using the face width in the WVC image can be degraded if the Z distance is large because of the reduction in the image resolution of the face in the NVC. Thus, the estimated Z distance was employed only for the initial Z distance estimation for auto-focusing, and the auto-focusing is performed based on calculated focus score by the focus mask (see Section 3.3). Then, the information about the estimated Z distance and the eye position of the WVC (Figure 6) is transferred to the NVC module using a TCP/IP socket program.

### 3.3. Panning, Tilting, and Focusing of NVC

In Figure 3b, the optical axes of the NVC and WVC in the proposed gaze tracking device are horizontally parallel. Thus, if the one of the cameras is moved, another camera is simultaneously moved in the same direction, and the parallel optical axes always remain parallel. Given this fact, panning and tilting of the device are performed as follows.

First, the gaze tracking device is panned and tilted according to the information it receives (the estimated Z distance and the eye position of the WVC). Then, auto-focusing of the NVC is performed.

**Figure 9.**
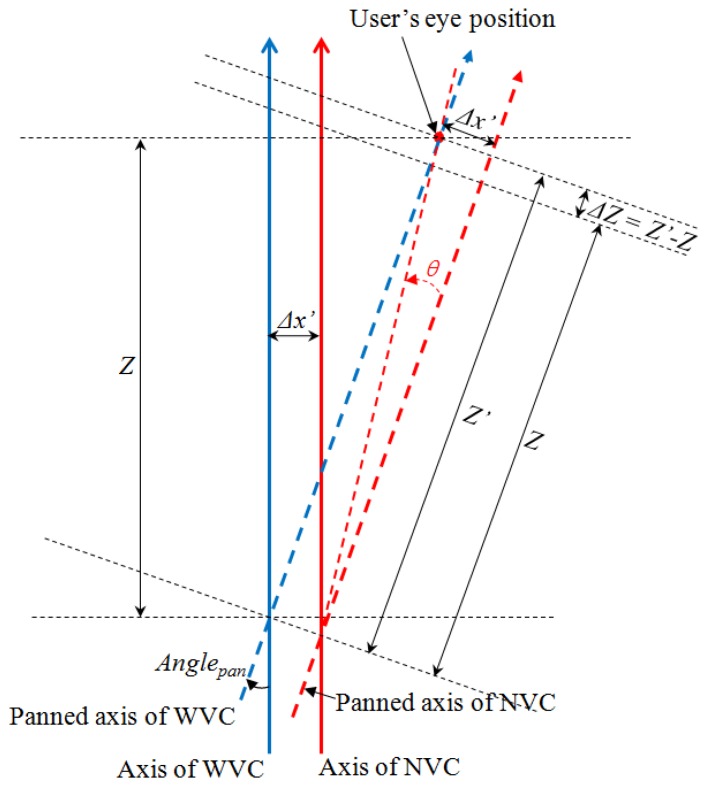
Analysis of *Angle_pan_* and *Offset_x_*.

For panning and tilting, each movement step of the motor was calculated with the following equation:

(6) $$\left[\begin{array}{c} {\text{Steps}}_{\text{pan}}\\ {\text{Steps}}_{\text{tilt}} \end{array}\right]=\left[\begin{array}{cc} \frac{1}{0.025522} & 0\\ 0 & \frac{1}{0.023864} \end{array}\right]\;\left[\left[\begin{array}{c} {\text{Angle}}_{\text{pan}}\\ {\text{Angle}}_{\text{tilt}} \end{array}\right]+\left[\begin{array}{c} {\text{Offset}}_{x}\\ {\text{Offset}}_{y} \end{array}\right]\right]$$

where 1/0.025522 and 1/0.02386 are determined on the basis of the motor specifications (between the angle and movement step of the motor). In Equation (6), *Angle_pan_* and *Angle_tilt_* are the rotation angles for panning and tilting, respectively. *Offset_x_* and *Offset_y_* are the steps for the additional rotation angles of the NVC in the horizontal and vertical directions, respectively, as shown in Figure 9.

The objective of panning and tilting is to capture an enlarged eye image with the NVC. Because the optical axes of the NVC and WVC have parallel horizontal (X) axes, as shown in Figure 3b, the distances (Δ*x*′ of Figure 9) between the two origins of the WVC and NVC in the horizontal axis are constant. Consequently, the additional rotation (*Offset_x_* and *Offset_y_*) of panning and tilting is required to capture an enlarged eye image with the NVC. *Angle_pan_* and *Angle_tilt_* are calculated using Equation (7) as follows:

(7) $$\left[\begin{array}{c} {\text{Angle}}_{\text{pan}}\\ {\text{Angle}}_{\text{tilt}} \end{array}\right]=\arctan\left[\begin{array}{c} \frac{x}{Z}\\ \frac{y}{Z} \end{array}\right]$$

where *x* and *y* are the coordinates of the predicted eye position of the WVC, and *Z* is the estimated Z distance. Additionally, in Equation (6), *Offset_x_* and *Offset_y_* are calculated using Equation (8):

(8) $$\left[\begin{array}{c} {\text{Offset}}_{x}\\ {\text{Offset}}_{y} \end{array}\right]=\arctan\left[\begin{array}{c} \frac{\Delta x\prime }{Z}\\ \frac{\Delta y\prime }{Z} \end{array}\right]+\left[\begin{array}{c} e_{x}\\ e_{y} \end{array}\right]$$

In Equation (8), Δ*x*′ and Δ*y*′ are the distances between the optical axes of the WVC and NVC in the horizontal (X of Figure 3b) and vertical (Y of Figure 3b) directions, respectively. As shown in Figure 9, *Offset_x_* of Equation (8) can be calculated by *arctan*(Δ*x*′/*Z′*) (as in *θ* of Figure 9). However, Δ*Z* (=*Z′* − *Z*) is considerably smaller than *Z* (or *Z′*), and *Z′* can consequently be assumed to be almost equal to *Z*. Thus, *Offset_x_* can be calculated by *arctan*(Δ*x*′/*Z*), with the additional term of *e_x_*. Similarly, *Offset_y_* can be calculated by *arctan*(Δ*y*′/*Z*), with the additional term of *e_y_*. In addition, because the Y- and Z-axes of the WVC shown in Figure 3b cannot be perfectly parallel to those (Y′ and Z′) of the NVC, respectively, the error terms of *e_x_* and *e_y_* are considered in Equation (8). Here, because the origin of the WVC of Figure 3b can be slightly translated in the direction of the Y axis compared to that of the NVC, Δ*y*′ is also considered in Equation (8).

After the panning and tilting based on Equation (6), the NVC captures the enlarged eye image. Then, a focus score is measured that determines whether the image is well focused or not. To calculate the focus score, the methods of Kang and Daugman were compared [31].

As shown in Figure 8, Daugman and Kang presented convolution kernels to measure whether the eye image is focused or not [31]. By using these methods, we measured a focus score that is normalized to a value from 0 to 1. The experimental results showed that the performance of Daugman's method was better than that of Kang's method. Because Kang's kernel is smaller than Daugman's, as shown in Figure 8, the calculated focus score by Kang's kernel is more affected by distracting objects, such as eyebrows and glasses frames, in the captured image of the NVC [31].

After calculating the focus score, if the score is greater than the predetermined threshold, the next stage of the proposed gaze tracking method is executed. However, if it is not, an additional step of auto-focusing is executed. That is, the focus lens is moved in the forward or backward direction on the basis of the measured focus score in the previous frame [31]. Then, the NVC captures the enlarged eye image again, and the focus score is measured in the captured image. These processes are iteratively carried out until the calculated focus score is greater than the threshold [31].

### 3.4. Detection of Pupil and SR in NVC Images

In the previous studies [1,3,4], a method called circular edge detection (CED) was used to the detect pupil area in an image that contains an eye. However, in their experimental image, the eye region fills a very large portion of the captured image, whereas in the proposed system, the eye region is comparatively smaller in the captured eye image from the NVC. Consequently, the processing time for the CED-based pupil detection becomes longer, and a significant error in the CED-based pupil detection can occur owing to the additional inclusion of an eyelash or and glasses frame compared to the cases of the previous studies [1,3,4].

To solve these problems, we used a pre-detection method of the pupil area by using the rapid eye detection (RED) method, which was proposed by Kim *et al.* [36]. Rapid eye detection is a method that can quickly detect the eye region in an image containing an eye. In the human eye, the intensities of the iris and its neighbors are significantly different [36], and this characteristic is exploited in the rapid eye detection method. The pre-detection method of the pupil area is carried out as follows.

Firstly, the image with a resolution of 1,600 × 1,200 pixels that is captured by the NVC is sub-sampled down to an image with a resolution of 400 × 300 pixels to reduce the processing time. Then, the rapid eye detection method is carried out. On the basis of the pupil area detected by the RED, the pupil center and four SR centers are detected as follows.

**Figure 10.**
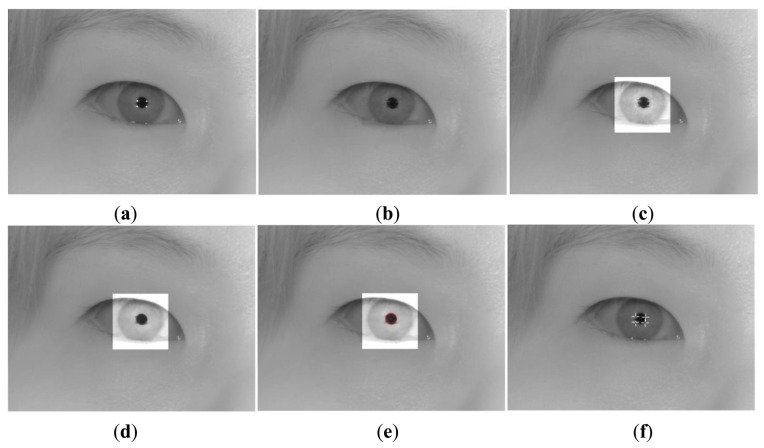
Steps for obtaining pupil center and four SR centers. (**a**) Original image. (**b**) Results of removing SRs. (**c**) Results of histogram stretching. (**d**) Results of the morphological operation. (**e**) Detection results of the pupil using CED. (**f**) Detection results of four SRs.

Figure 10a shows an image that was captured by the NVC. To calculate the pupil center, the corneal SRs around the pupil are removed, as shown in Figure 10b. Then, we performed histogram stretching to enhance the distinct difference in brightness between the pupil and the iris, as shown in Figure 10c. Next, a gray morphological operation [37] using a 7 × 7 pattern is performed to smooth the removed region of the SRs, as shown in Figure 10d. The pattern has a circular shape similar to the pupil, and the removal of the SR, which has a bright gray color, can be performed by iterating the procedure of erosion and dilation. Subsequently, the CED is performed, and its center is regarded as the pupil center, as shown in Figure 10e. The red circle is a result of the CED, and red point inside the red circle is the pupil center. On the basis of the pupil center, the search region for detecting the SR is defined. Within the search region, the four SRs are detected by binarization, component labeling, and calculating the geometric center, as shown in Figure 10f [3,5,37].

### 3.5. User-Dependent Calibration

As shown in Figure 1, in the proposed system, user-dependent calibration should be performed before calculating the gaze position. As explained in Section 3.2, the actual facial width (*A* of Equation (5)) is obtained at the stage of initial user-dependent calibration. With *A*, *a* (facial width in the image), and *f* of Equation (5), the Z distance between the user and gaze tracking device can be successively obtained by using Equation (5). On the basis of the Z distance and the eye position in the WVC, accurate panning, tilting, and focusing of the device are carried out, as explained in Section 3.3. As shown in the previous studies [1,3,4], the pupillary axis is different from the gaze axis, and the angle between these two axes is called kappa. Consequently, the user-dependent calibration for obtaining kappa is performed by gazing at the center of the TV screen.

### 3.6. Calculating Final Gaze Position

**Figure 11.**
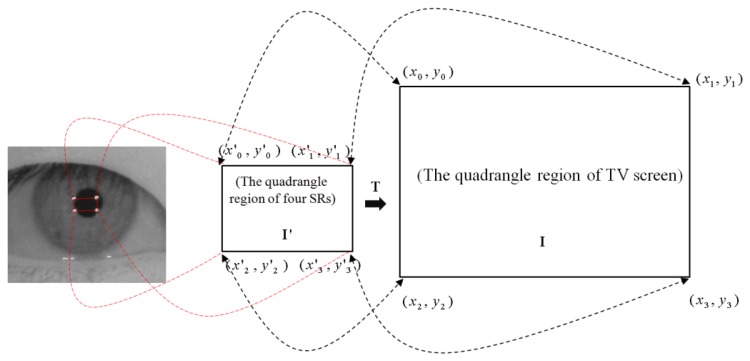
Mapping relationship between TV screen region and quadrangle region of four SRs [1,3,5,6].

With the detected pupil center and four SR centers of Section 3.4, a geometric transformation is performed to calculate the gaze position, as shown in Figure 11.

In the proposed method, the four NIR illuminators are attached to the four corners of the TV, as shown in Figure 2. Thus, the four SRs created by the illuminators are shown on the cornea, as shown in Figure 11. Then, the four center points of the four SRs, denoted by (*x′_0_*, *y′_0_*), (*x′_1_*, *y′_1_*), (*x′_2_*, *y′_2_*), and (*x′_3_*, *y′_3_*), created by the illuminators can be mapped onto the four corners of the TV, denoted by (*x_0_*, *y_0_*), (*x_1_*, *y_1_*), (*x_2_*, *y_2_*), and (*x_3_*, *y_3_*), respectively. These relationships can be represented by the geometric transformation shown in Equation (9) [1,3,5,6,37,38]. The geometric transformation has 8 unknown parameters (*a*, *b*, *c*, *d*, *m*, *n*, *p*, and *q*) [37]:

(9) ITI'[x0x1x2x3y0y1y2y300000000]=[abcdmnpq00000000]⋅[x0′x1′x2′x3′y0′y1′y2′y3′x0′y0′x1′y1′x2′y2′x3′y3′1111]

Elements *a*, *b*, *c*, *d*, *m*, *n*, *p*, and *q* of **T** can be calculated by multiplying **I** with **I′**^−1^.

When the user is watching TV, the detected pupil center (*x′_g_*, *y′_g_*) in the eye image is mapped to the TV display by using the following equation [1,3,5,6,38]:

(10) $$\left[\begin{array}{c} x_{g}\\ y_{g}\\ 0\\ 0 \end{array}\right]=\left[\begin{array}{cccc} a & b & c & d\\ m & n & p & q\\ 0 & 0 & 0 & 0\\ 0 & 0 & 0 & 0 \end{array}\right]\left[\begin{array}{c} x_{g}^{\prime }\\ y_{g}^{\prime }\\ x_{g}^{\prime }y_{g}^{\prime }\\ 1 \end{array}\right]+\left[\begin{array}{c} k_{x}\\ k_{y}\\ 0\\ 0 \end{array}\right]$$

In Equation (10), *k_x_* and *k_y_* are the values that compensate for kappa (see Section 3.5). From Equation (10), the final gaze point (*x_g_*, *y_g_*) is calculated. This geometric transformation based method is different from the mentioned cross-ratio based one because the latter uses the vanishing points [39].

## 4. Experimental Results

Two experiments were performed to measure the performance of the proposed gaze tracking system. The first one is the measurement of the accuracy of the panning and tilting mechanisms. The second one is the measurement of the gaze tracking accuracy. All of the experiments were performed using a desktop computer with a 3.2-GHz CPU and 4 GB of RAM.

### 4.1. Accuracy of Panning and Tilting Mechanisms

**Figure 12.**
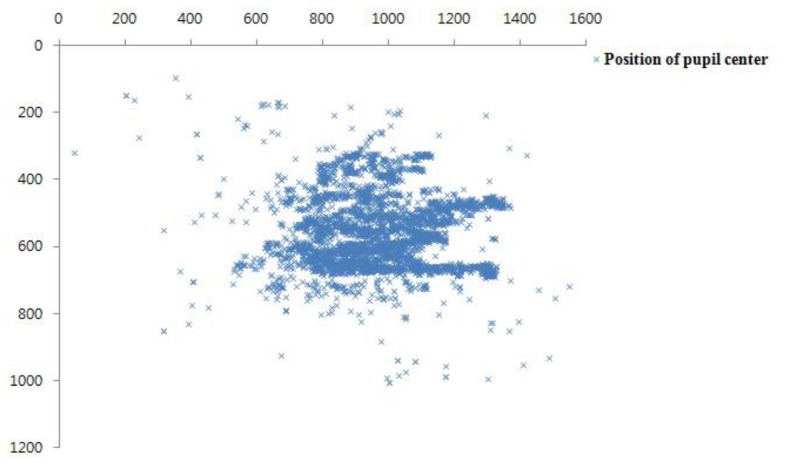
Positions of pupil centers of 4,200 NVC images after panning and tilting.

As explained in Section 3.3, the proposed gaze tracking system is operated on the basis of panning and tilting mechanisms. For continuous gaze tracking, the eye should always be included in the captured image from the NVC. Ideally, the position of the pupil center should be located at the center of the NVC image. Therefore, we plotted the positions of the pupil center that were obtained from 4,200 images (of 10 users) from the NVC by using our panning and tilting mechanisms, as shown in Figure 12. Because the image resolution of the NVC is 1,600 × 1,200 pixels, the center position of the NVC image is (800, 600). Experimental results showed that the average x and y positions of pupil centers of the data were 970.97 (the standard deviation of 174.44) and 559.15 (the standard deviation of 110.27), respectively. If there is no error in the system from panning and tilting, the averages of the x and y positions should be 800 and 600, respectively, with standard deviations of almost 0. In the proposed system, however, the panning and tilting mechanisms have a small amount of error because there are errors in the predicted x and y positions of the eye in the WVC image. This is because the size of the eye in the captured image from the WVC is significantly smaller, as shown in Figure 6. In addition, the Z distance estimation has a degree of error due to the small image resolution of face in the WVC, as shown in Figure 6. That is because the panning and tilting angles are determined on the basis of the x and y positions of the eye and the Z distance measured in the WVC, as shown in Equation (7). However, the pupil regions in all of the 4,200 images are successfully included in the NVC image (with no error) after panning and tilting, and further processing for the gaze tracking shown in Figure 1 can proceed in all of the 4,200 images.

### 4.2. Gaze Tracking Accuracy

**Figure 13.**
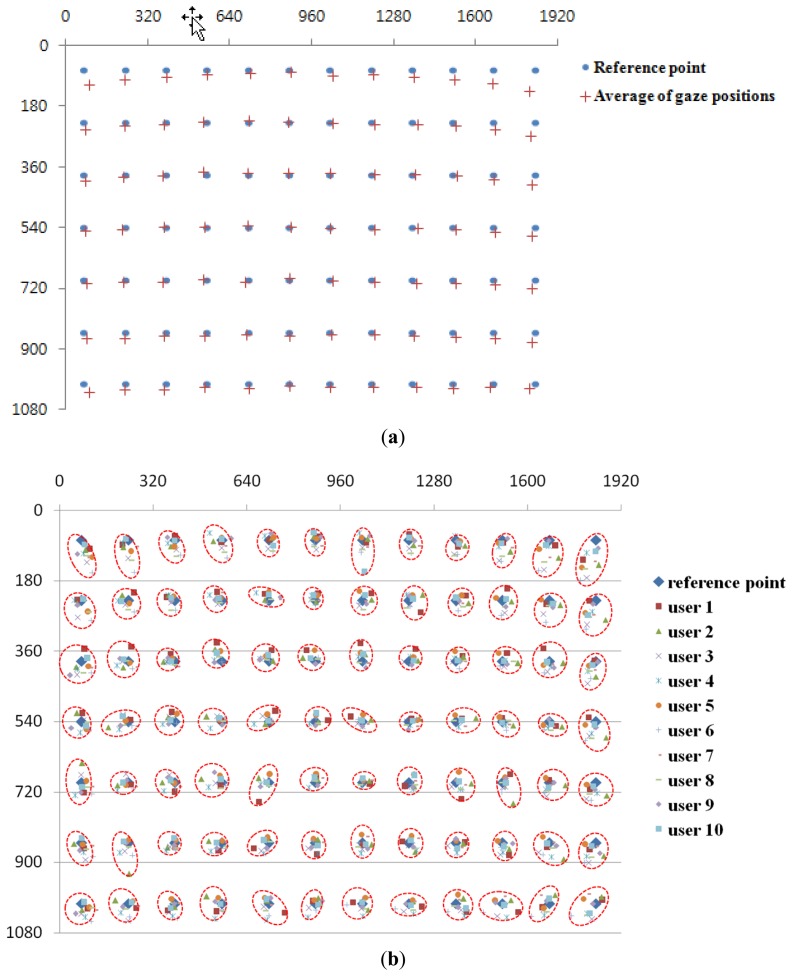
Results of average gaze positions of 10 users. (**a**) Average gaze positions of 10 users with five trials. (**b**) Average gaze positions of five trials per each user.

In many previous studies [1,4,5,10], researchers measured the gaze tracking accuracy by using only nine reference points on the display. In order to measure the accuracy of gaze tracking more intensively, we made measurements using 84 reference points, as shown in Figure 13. A total of ten subjects participated in this experiment. They sat on a chair at a Z distance of 1.8–2.2 m from the TV display with the sitting range of ±20 cm based on center in the horizontal direction. In addition, they naturally moved their heads (less than about ±20 cm in X, Y, and Z directions, respectively) and eyes during the experiments. The sitting heights of ten subjects are from about 115 to 145 cm. Each subject tried to look at the 84 reference points five times.

The results are shown in Figure 13 and Table 1. The disparity between the reference position and the calculated gaze position can be measured, and the errors (with standard deviation) of gaze tracking in Table 1 can be calculated by using the disparities and the Z distance. From the results, the average gaze error was found to be approximately ±0.737°, which was smaller than the error found in previous studies [1,3,5].

In Table 1, the average gaze tracking errors of users 1 and 2 are relatively larger than those of other people. This is caused by the incorrect user-dependent calibration. As explained in Section 3.5, each user should gaze at the center position of display in the initial calibration stage, from which the individual kappa angle is obtained. In case of users 1 and 2, they did not gaze at the accurate center position in the calibration stage, which increased the gaze tracking error, consequently.

**Figure 14.**
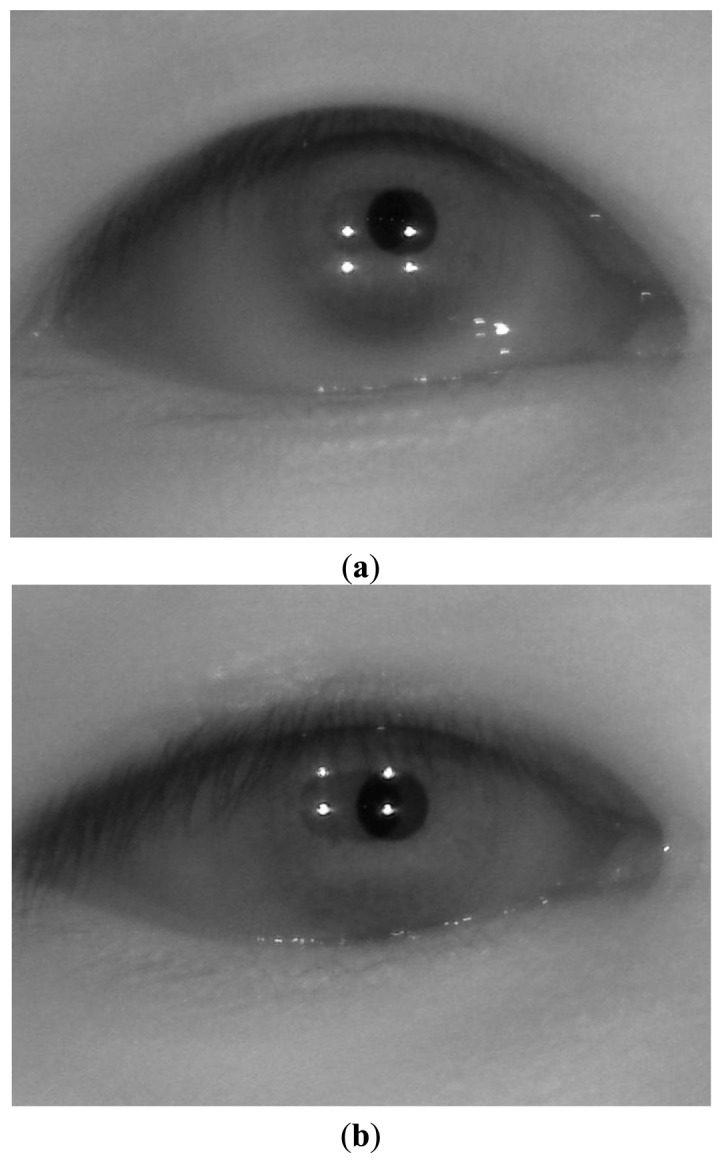
Locus of pupil centers. (**a**) In case of gazing at the left upper-most to right upper-most position. (**b**) In case of gazing at the left lower-most to right lower-most position.

In Figure 13a, the calculated gaze positions have the tendency of parabolic shape in horizontal direction. In details, the gaze error of vertical direction at the middle upper-most position is smaller than that at the left upper-most or right upper-most position. This phenomenon is caused by the followings. As shown in the Figure 2, our gaze tracking system is positioned below the user's face, and the user gazes at the display above the gaze tracking system. So, even if the user's eye is rotated in the horizontal direction while gazing at the position of left upper-most to right upper-most position, the locus of pupil center in the NVC images is not the horizontal line but the parabolic shape. Consequently, the gaze error of vertical direction at the middle upper-most position is smaller than that at the left upper-most or right upper-most position. This phenomenon becomes severe when a user gazes at the left upper-most to right upper-most position (the locus of pupil center which is shown by the red dotted line of Figure 14a compared to the left lower-most to right lower-most position, the locus of the pupil center which is shown by the red dotted line of Figure 14b) because the angle disparity between the user's line of sight and the optical axis of the NVC to the user's eye becomes larger in the former case. Figure 13b shows the average gaze position of five trials of each user.

For the next experiment, we measured the processing time of the proposed method shown in Figure 1. The experimental results showed that the proposed gaze tracking system could be operated at a speed of 5∼10 frames/s.

**Figure 15.**
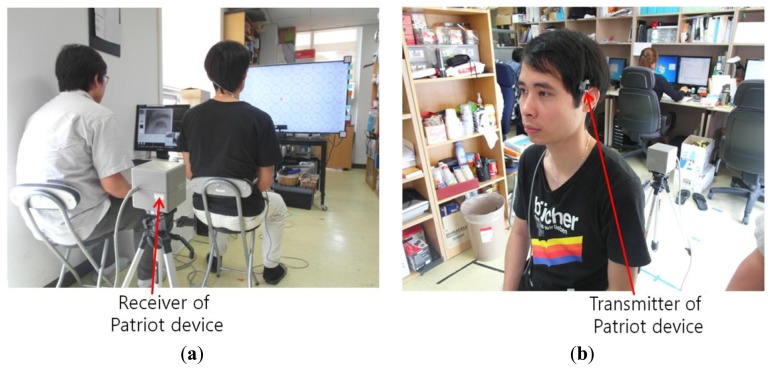
Examples of experimental setup. (**a**) Image of back side. (**b**) Image of front side.

We performed the additional experiments. In these experiments, each subject attached Polhemus position tracking sensor (Patriot sensor [40]) on his left temple (the area between left eye and ear) in order to measure the maximum velocity of head movement (which is allowed for the operation of our gaze tracking system) as shown in Figure 15.

A total of 10 subjects participated in the experiments, and each subject gazed at 84 points on the display of 60 inches (the resolutions of 1,920 × 1,080 pixels). In order to measure the effect caused by the sitting position, each subject repeated this procedure at five different sitting positions: left (about 20 cm from the middle), middle, right (about 20 cm from the middle), closer (about 20 cm from the middle), and farther (about 20 cm from the middle) positions from the display, respectively. Here, the middle position means the about center position of the display with the Z distance of about 200 cm from the display.

In the Table 2, the average and standard deviation of head movement velocity of each subject are included. In addition, the maximum average velocity of head movement of each subject is included. From them, we can know that the maximum velocities of head movement (which is allowed for the operation of our gaze tracking system) are 2.887 cm/s, 3.142 cm/s, and 1.305 cm/s (in X, Y, and Z directions), respectively.

**Figure 16.**
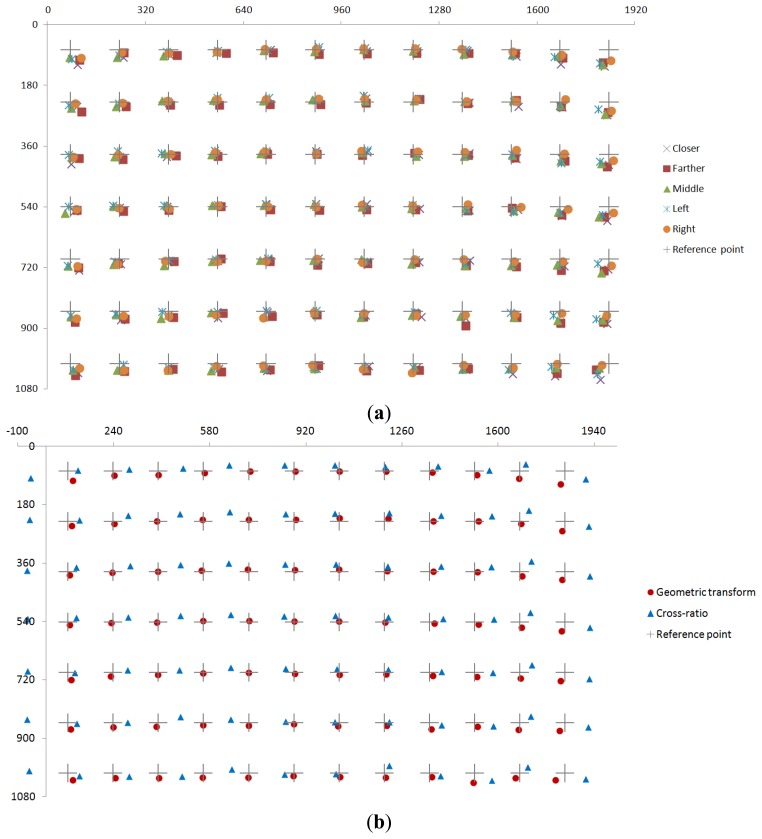
Results of average gaze positions of 10 users. (**a**) Average gaze positions of 10 users according to five sitting positions. (**b**) Average gaze positions of 10 users in case of geometric transform and cross-ratio methods, respectively.

In Tables 3, 4, 5 and 6 and Figure 16, gaze tracking errors are included. The disparity between the reference position and the calculated gaze position is measured, and the Table 4 shows this disparity by the unit of pixel or mm. And the errors (with standard deviation) of gaze tracking in Table 3 are calculated by using this disparity and the Z distance.

We compared the gaze tracking error by our geometric transform-based method to that obtained by the cross-ratio-based one. In previous research [39], they used the cross-ratio-based method using vanishing points for calculating the gaze position. Since our system requires a user to gaze at one center position in the display in the initial calibration stage, we apply our calibration scheme (gazing at only one center position) to the cross-ratio-based method for fair comparison. As shown in Tables 3, 4, 5 and 6, and Figure 16b, we can confirm that the proposed method based on the geometric transform outperforms the cross-ratio-based method. In addition, we can find that the gaze tracking accuracy of our method is not much affected by the sitting positions, as shown in Tables 5 and 6, and Figure 16a. The gaze tracking error of our method in Tables 3 and 5 is little larger than that in Table 1 because the head movement of each subject in Tables 3 and 5 was bigger than that in Table 1.

From the Tables 5 and 6, and Figure 16a, we can confirm that the performance of our gaze tracking system is not much affected by the different gaze-angles and sitting (head) positions.

## 5. Conclusions

We have proposed a novel gaze tracking system for the interface of an intelligent TV. A gaze tracking device with a WVC and an NVC was designed and operated on the basis of panning and tilting mechanisms. The proposed system could stably track the gaze of the user at a distance from 1.8 to 2.2 m. In the experimental results, the average error of the gaze tracking system was measured as ±0.737°∼±0.775° and the processing speed was 5∼10 frames/s. Moreover, by using panning and tilting mechanisms, the proposed system allows a wider range of freedom of head movement than has been possible in previous studies or commercial products.

This ‘eye-gaze’ information can be used for the menu selection of the user interface program of the large display such as smart TV and digital signage, *etc*. In addition, it can be used for monitoring the user's interest among the various contents displayed or the audience rating of TV watchers. And it can be used for the intelligent interface for the handicapped or patient, *etc*.

Since these functionalities cannot be executed just once but are continuously performed, especially when the user watches the TV, ‘eye-gaze’ should be tracked in the successive frames. If the gaze detection error is large, it is difficult to select from a small menu. Consequently, the menu size increases and the number of possible menus on the TV screen is reduced, which makes it inconvenient for users to select from various menus. In addition, this error can cause the user to select the menu he does not want, which can increase the transaction time and the user's inconvenience, so a lower gaze detection error is required. We can confirm that a total of 264 menus (22 × 12) on a screen can be selected by our gaze tracking system considering our gaze error of ±0.775° at the Z distance of 2.2 m on a TV of 60 inches in size. In future work, we would like to research a method using an additional device such as an NIR-based distance measuring device or a high-cost laser scanner in order to measure the Z distance more accurately.

## Figures and Tables

**Table 1.** Errors of proposed gaze tracking method.

**User**	**Average of Gaze Tracking Error (Standard Deviation)**
User 1	±0.937° (0.212)
User 2	±0.946° (0.140)
User 3	±0.735° (0.099)
User 4	±0.706° (0.102)
User 5	±0.627° (0.068)
User 6	±0.821° (0.117)
User 7	±0.635° (0.065)
User 8	±0.761° (0.075)
User 9	±0.690° (0.030)
User 10	±0.514° (0.051)
Total average	±0.737° (0.096)

**Table 2.** Measured velocity of head movement of each subject (unit: cm/s).

**Methods**	**Average and Standard Deviation of Head Movement Velocity in All the Sitting Positions**						**Maximum Average Velocity of Head Movement**		

	**X Axis**		**Y Axis**		**Z Axis**		**X Axis**	**Y Axis**	**Z Axis**
			
**Subject**	**Avg.**	**Std.**	**Avg.**	**Std.**	**Avg.**	**Std.**			
User 1	1.014	0.582	1.052	0.660	0.536	0.198	2.024	2.175	0.852
User 2	0.800	0.491	0.880	0.562	0.423	0.176	1.608	1.783	0.645
User 3	1.361	0.774	1.481	0.900	0.761	0.301	2.671	2.956	1.179
User 4	1.692	0.808	1.883	0.925	0.942	0.372	**2.887**	**3.142**	**1.305**
User 5	1.155	0.697	1.231	0.787	0.586	0.263	2.299	2.513	0.982
User 6	1.200	0.614	1.313	0.730	0.637	0.236	2.088	2.356	0.897
User 7	1.160	0.565	1.155	0.595	0.637	0.182	2.109	2.144	0.899
User 8	1.097	0.490	1.187	0.589	0.564	0.195	1.780	1.993	0.753
User 9	0.858	0.496	0.908	0.583	0.495	0.206	1.702	1.907	0.834
User 10	1.187	0.618	1.273	0.713	0.635	0.274	2.122	2.353	0.935
Avg.	1.1524	0.6135	1.2363	0.7044	0.6216	0.2403	2.129	2.3322	0.9281

**Table 3.** Comparisons of gaze tracking error by our geometric transform-based method to that by cross-ratio based one in each subject—(1) (unit: degree).

**Method**	**Geometric Transform**		**Cross-Ratio**	
		
**Subject**	**Avg.**	**Std.**	**Avg.**	**Std.**
User 1	±0.917	0.562	±1.021	0.602
User 2	±0.828	0.521	±1.954	1.229
User 3	±0.651	0.698	±1.720	1.319
User 4	±0.550	0.358	±1.734	1.140
User 5	±0.728	0.434	±1.670	1.090
User 6	±0.620	0.375	±1.321	0.802
User 7	±0.926	0.480	±1.625	0.967
User 8	±0.662	0.487	±1.241	1.184
User 9	±1.036	0.556	±2.590	2.030
User 10	±0.835	0.514	±1.567	1.016
Avg.	±0.775	0.499	±1.644	1.138

**Table 4.** Comparisons of gaze tracking error by our geometric transform-based method to that by cross-ratio based one in each subject—(2) (unit: pixel or mm).

**Method**	**Geometric Transform**				**Cross-Ratio**			
	
	**Error (pixel)**		**Error (mm)**		**Error (pixel)**		**Error (mm)**	
				
**Subject**	**X axis**	**Y axis**	**X axis**	**Y axis**	**X axis**	**Y axis**	**X axis**	**X axis**
User 1	37.11	20.11	25.71	13.93	40.27	22.66	27.90	15.69
User 2	21.03	31.97	14.57	22.15	89.56	28.44	62.04	19.70
User 3	20.91	19.34	14.48	13.40	74.66	24.04	51.72	16.65
User 4	17.23	15.27	11.93	10.58	69.55	28.42	48.18	19.68
User 5	27.04	17.76	18.73	12.31	74.17	21.75	51.38	15.07
User 6	21.78	16.47	15.09	11.41	53.73	25.79	37.22	17.87
User 7	30.08	31.52	20.83	21.84	74.82	20.42	51.83	14.14
User 8	22.57	18.73	15.64	12.97	50.61	23.07	35.05	15.98
User 9	39.20	26.20	27.15	18.15	114.34	40.07	79.21	27.76
User 10	27.18	25.47	18.83	17.65	68.27	25.28	47.29	17.51
Avg.	26.41	22.28	18.30	15.44	71.00	25.99	49.18	18.01

**Table 5.** Comparisons of gaze tracking error by our geometric transform-based method to that by cross-ratio based one in each sitting position—(1) (10 subjects) (unit: degree).

**Method**	**Geometric Transform**		**Cross-Ratio**	
		
**Position**	**Avg.**	**Std.**	**Avg.**	**Std**
Middle	±0.835	0.539	±1.692	1.175
Left	±0.746	0.446	±1.549	1.538
Right	±0.682	0.435	±1.708	1.155
Closer	±0.755	0.480	±1.775	1.291
Farther	±0.859	0.683	±1.497	1.069
Avg.	±0.775	0.517	±1.644	1.246

**Table 6.** Comparisons of gaze tracking error by our geometric transform-based method to that by cross-ratio based one in each sitting position—(2) (10 subjects) (unit: pixel or mm).

**Method**	**Geometric Transform**				**Cross-Ratio**			
	
	**Error (pixel)**		**Error (mm)**		**Error (pixel)**		**Error (mm)**	
				
**Position**	**X Axis**	**Y Axis**	**X Axis**	**Y Axis**	**X Axis**	**Y Axis**	**X Axis**	**Y Axis**
Middle	30.36	21.58	21.03	14.95	75.03	23.79	51.97	16.48
Left	25.41	22.00	17.60	15.24	65.42	28.15	45.32	19.50
Right	22.37	20.09	15.50	13.91	74.34	26.61	51.50	18.43
Closer	21.28	21.36	14.74	14.80	70.49	24.43	48.83	16.92
Farther	32.64	26.40	22.61	18.29	69.71	26.98	48.29	18.69
Avg.	26.41	22.28	18.30	15.44	71.00	25.99	49.18	18.01
